# Demystifying heart failure with a preserved ejection fraction: what you need to know

**DOI:** 10.3399/bjgp24X736396

**Published:** 2024-03-01

**Authors:** Ian Loke, Sotiris Antoniou, Roselin Boramakot, David Walters, Ahmet Fuat

**Affiliations:** Department of Cardiovascular Sciences, University Hospitals of Leicester, Leicester.; Cardiovascular Medicine, St Bartholomew’s Hospital, Barts Health NHS Trust, London.; Boehringer Ingelheim UK and Ireland, Bracknell.; Boehringer Ingelheim UK and Ireland, Bracknell.; Carmel Medical Practice, Darlington.

## Introduction

Over 900 000 people in the UK have a heart failure (HF) diagnosis, with its incidence and prevalence highest among older people — the average age at diagnosis is 77 years.^1^ HF is associated with frequent hospitalisations and reduced health-related quality of life and, therefore, represents a considerable health burden for patients and the NHS as a whole.^2^^,^^3^

This editorial will highlight the vital role that primary care has in the diagnosis and management of HF, particularly the form of HF where ejection fraction (EF) is preserved.

## Heart failure categories

There are three main categories of HF, as defined by the European Society of Cardiology (ESC) 2021 guidelines: HF with reduced ejection fraction (HFrEF), where left ventricular ejection fraction (LVEF) is ≤40%; HF with mildly reduced ejection fraction (HFmrEF), where LVEF is 41%–49%; and HF with preserved ejection fraction (HFpEF), where LVEF is ≥50%.^4^ Approximately half of all HF is thought to be HFpEF, a patient population that currently has a large unmet clinical need. This is due to the challenges of making an accurate diagnosis, availability of HF services that manage HFpEF, and a historical lack of efficacious pharmacotherapy.^5^

**Figure 1.**
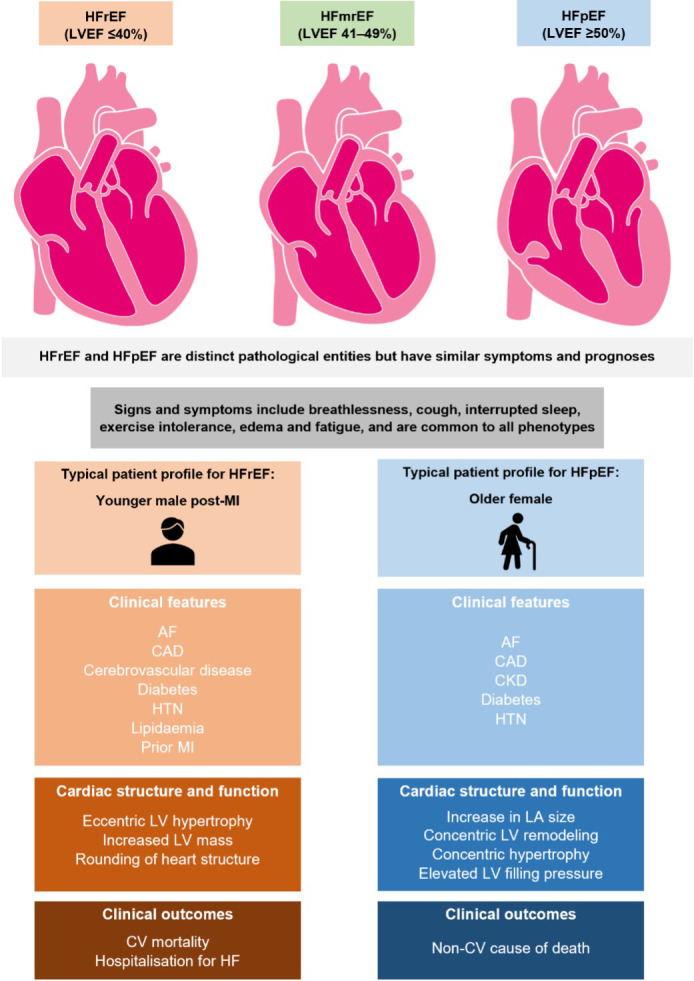
The different categories of heart failure, their associated symptoms, and typical patient profiles. Figure adapted from Zhou *et al*^9^ under the Creative Commons Attribution Licence (CC-BY), with additional information.^4^^,^^6^^–^^13^ AF = atrial fibrillation. CAD = coronary artery disease. CKD = chronic kidney disease. CV = cardiovascular. HF = heart failure. HFmrEF = heart failure with mildly reduced ejection fraction. HFpEF = heart failure with preserved ejection fraction. HFrEF = heart failure with reduced ejection fraction. HTN = hy per ten sion. LA = left atrial. LV = left ventricle. LVEF = left ventricular ejection fraction. MI = myocardial infarction.

Figure 1 illustrates the different classifications of HF; while some key risk factors overlap, the underlying pathophysiological mechanisms differ between the classifications. When considered alongside the signs and symptoms of HF, the typical patient profiles may aid with the identification of patients with suspected HF presenting to primary care.^4^^,^^6^^–^^8^

## Making a diagnosis

The 2018 National Institute for Health and Care Excellence (NICE) and 2021 ESC guidelines provide HF diagnostic and treatment algorithms.^1^^,^^4^ NICE guidelines recommend taking a detailed patient history and performing a clinical examination where chronic HF is suspected; this is often triggered by patients presenting to primary care with the common signs and symptoms of HF (as outlined in Figure 1). As well as diagnosis in primary care, patients can be diagnosed in hospital during an episode of decompensated HF. In the primary care setting, it is important to exclude other causes of breathlessness as well as other conditions that mimic HFpEF (for example, lung disease, anaemia, arrhythmia, valve disease, obesity, and deconditioning).^4^ After presenting with signs and symptoms and taking a thorough history, the formal path to diagnosis of HF continues with the measurement of N-terminal pro-B-type natriuretic peptide (NT-proBNP) levels; an electrocardiogram (ECG) and other blood tests are also advisable. If NT-proBNP levels are elevated, specialist clinical assessment follows, which includes echocardiography, whereby EF is estimated and HF is categorised. In the case of HFpEF in particular, in addition to the steps outlined above, cardiologists will consider elements of echocardiographic evidence indicative of diastolic impairment.^4^ NICE guidelines also recommend that additional diagnostic tests should be considered, such as chest X-ray, blood tests, urinalysis, and peak flow or spirometry.^1^

## Management considerations

In addition to the differences between HFrEF and HFpEF from an aetiological perspective, there are also differences in evidence-based therapies for these populations. As covered in the 2021 ESC guidelines (and the 2023 focused update on recent clinical trials),^4^^,^^14^ there are several classes of drugs that have shown efficacy in patients with reduced but not preserved EF. These include angiotensin-converting enzyme inhibitors, angiotensin receptor blockers, beta-blockers, mineralocorticoid receptor antagonists, and angiotensin receptor-neprilysin inhibitors. Sodium-glucose co-transporter 2 (SGLT2) inhibitors have demonstrated efficacy across the left ventricular EF spectrum, in both HFrEF and HFpEF.^4^^,^^14^ The mainstay of treatment in HFpEF has therefore evolved from the core principles of focusing on symptomatic relief and with diuretics alongside the multifactorial approach to the management of cardiovascular (hypertension and atrial fibrillation [AF]) and non-cardiovascular (diabetes, obesity, obstructive sleep apnoea, iron deficiency anaemia, and chronic kidney disease [CKD]) comorbidities. Because of the complex nature of both forms of HF, it is recommended by current NICE guidance that patients with HF are managed by a specialist HF multidisciplinary team, alongside and supporting primary care.^1^ The provision of care by community HF nursing teams for the patient with HFpEF is inequitable in the country and it is important that secondary care HF services are available for patients who are not responding to standard therapy.

## The importance of coding

Accurate coding of HFpEF is essential and allows for better targeted management at an earlier stage of the disease, avoidance of non-evidence-based therapy, and even recruitment into clinical trials.^15^ Terminology such as left ventricular systolic dysfunction, left ventricular diastolic dysfunction, hypertensive cardiomyopathy, and left- or right-sided HF can complicate the classification of HF. This explains why current guidelines focus on LVEF threshold phenotypes.^4^^,^^15^ In a 2016 study of 300 000 adults with HF in the UK Clinical Practice Research Database, the five Read codes indicating HFpEF or diastolic HF were rarely used (1.26% combined prevalence) in general practice records,^16^ despite evidence that approximately half of primary care HF diagnoses can be attributed to HFpEF.^7^

Coding is usually done by the GP, assisted by clinical pharmacists and non-clinical administrative staff. Non-clinical administrative staff often code directly from hospital correspondence or outpatient clinical letters, suggesting that issues in coding could arise at the secondary/primary care interface. In addition to a need to upskill the wider primary care team to enable efficient and correct HF coding, there are wider issues surrounding why diagnoses are not always coded in primary care, such as the time required, diagnostic uncertainty, and GP experience. Better HF coding within primary care would improve the management of HFpEF, reduce inefficient prescribing, and help guide eligible patients into HFpEF clinical studies.^16^ Many patients with HF often present with multifactorial comorbidities that require treatment; this can also be facilitated by early identification.

## Conclusions

In the UK, HF is associated with high rates of hospitalisation and significant morbidity and mortality, representing a large and growing burden on the UK healthcare system. The different phenotypes of HF can present in the same manner but have different pharmacotherapy treatment options. HFpEF is a distinct entity within the HF spectrum, and it is important to identify and diagnose this early in patients with high-risk comorbidities (previous myocardial infarction, ischaemic heart disease, AF, diabetes, hypertension, and CKD). Primary care has an important role in diagnosing and managing this patient population, and having NT-proBNP measurement and echocardiography to back up clinical acumen is essential. An accurate diagnosis of the type of HF phenotype, be it HFrEF or HFpEF, will allow prompt initiation of appropriate lifesaving evidence-based therapy. Careful attention to HF coding in primary care, particularly HFpEF, will ensure accurate patient identification after diagnosis, setting the patient on the right path for treatment and disease management.
